# Supplementary material for: Assessing the impact of imperfect adherence to artemether-lumefantrine on malaria treatment outcomes using within-host modelling
Source: Nat Commun. 2017 Nov 9;8:1373. doi: 10.1038/s41467-017-01352-3 (PMC5680187; doi:10.1038/s41467-017-01352-3)
Supplement: Supplementary file 1 — Supplementary Information [file 41467_2017_1352_MOESM1_ESM.pdf]

Supplementary Materials

Supplementary Table 1: The parameter values that provided the best model fit to the malaria therapy data. The model equations are shown in the main text.

Parameter(s)	Value	Description
$m(t)$	Drawn from $\mathrm{N}(\mu = 16, \sigma = 8.7)$, truncation points: (1,35). Correlation strength $g = 0.63$.	Stochastic growth rate, positively correlated in time
$(\kappa_c, \kappa_v, \kappa_m)$	(3,3,1)	
$f(t)$	$f(t) = \lambda^{(t-4)}(t-4), \lambda = 0.9996$	Governs duration of EVSR memory
P_v^*	10100	Controls strength of the EVSR
k_m	0.021	
P_m^*	P_m^* / k_m drawn from Gompertz distribution $(\alpha = 0.0311, \theta = 0.0004)$	Controls the general adaptive response
k_c	0.164	
P_c^*	P_c^* / k_c drawn from $\ln N(\mu, \sigma)$, $(\mu = \ln 10^{4.79}, \sigma = 1.2)$. Truncated at $10^{5.5}$.	Controls the innate response
β	0.01	Controls max. efficacy of the general adaptive immunity
C	1	Regulates the growth rate of the general adaptive immunity

Supplementary Figure 1: An illustration of the memory of the effective var-specific immune response. The upper panel shows how the memory of the immune response increases over the course of the infection. The uppermost line (red) shows the upper limit of the sum given in Eq. 4. The value of the lower limit is determined by the function $f(t) = (t-4)\lambda^{(t-4)}$. Three candidates for the lower limit are shown for $\lambda = 0.9996$ (blue line), $\lambda = 0.999$ (orange line), and $\lambda = 0.998$ (green line). The arrows indicate the duration of the memory after 360 days in the intermediate case. The lower panel shows one run of our within-host model. The grey window, corresponding to the arrows in the upper panel, contains the parasitaemia used to calculate the EVSR after 360 days.

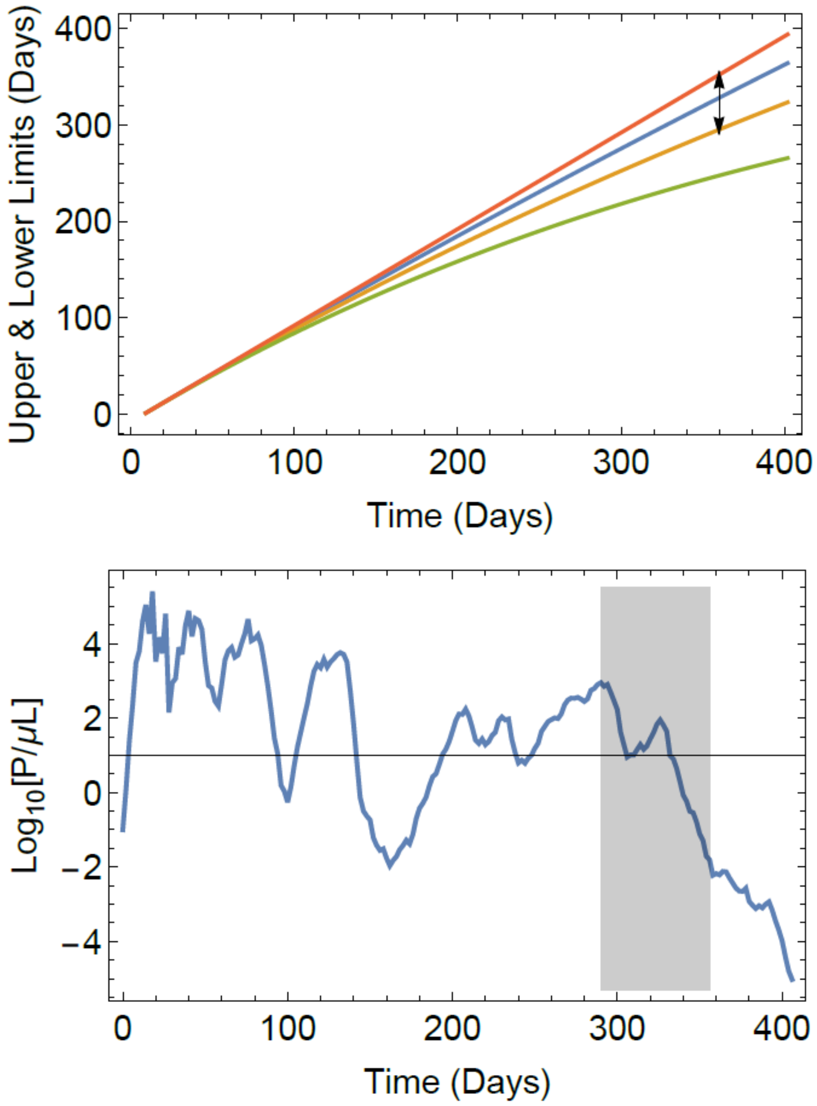

Supplementary Figure 2: Simulated blood concentrations for artemether and lumefantrine, including population-level variation. The black lines represent the median concentrations at each time point, as well as the 5th and 95th centiles. We also show the blood concentrations for the drugs' active metabolites (dihydroartemisinin and desbutyl-lumefantrine respectively). These plots were generated using the body weight distribution utilised to fit the PD model.

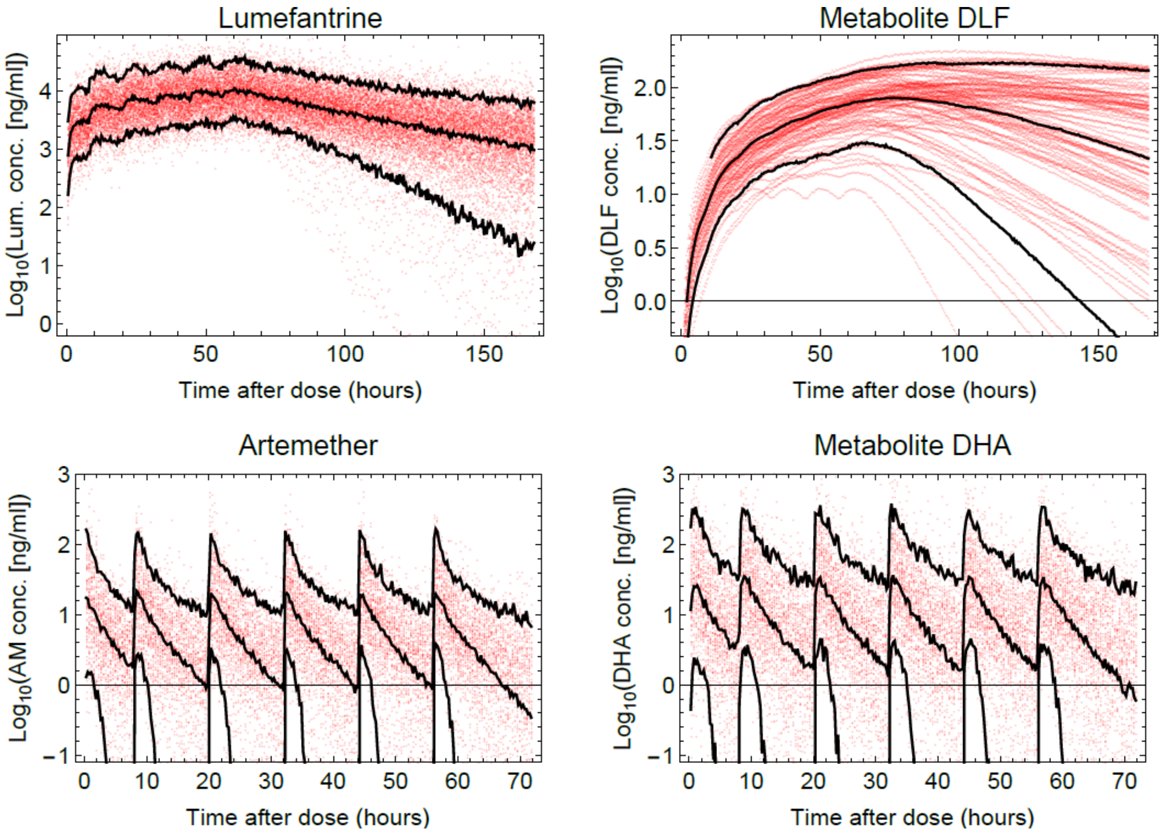

Supplementary Figure 3: The combination of the parasitaemia and drug models. Panel A shows the treated (blue line) and untreated (yellow line) parasitaemia for the same simulated infection. The peak of the untreated infection is used to determine the pyrogenic threshold (purple, horizontal line), as described in the Supplementary Methods. Panel B shows the timing of the first dose, indicated by the green, vertical line. The light blue, vertical line is used to determine the parasite density 24 hours later. Log-linear interpolation is used to determine whether the patient is parasite-positive at this point, as shown by the black line in panel C. Finally, in panel D we add the microscopy threshold (the red, dashed line).

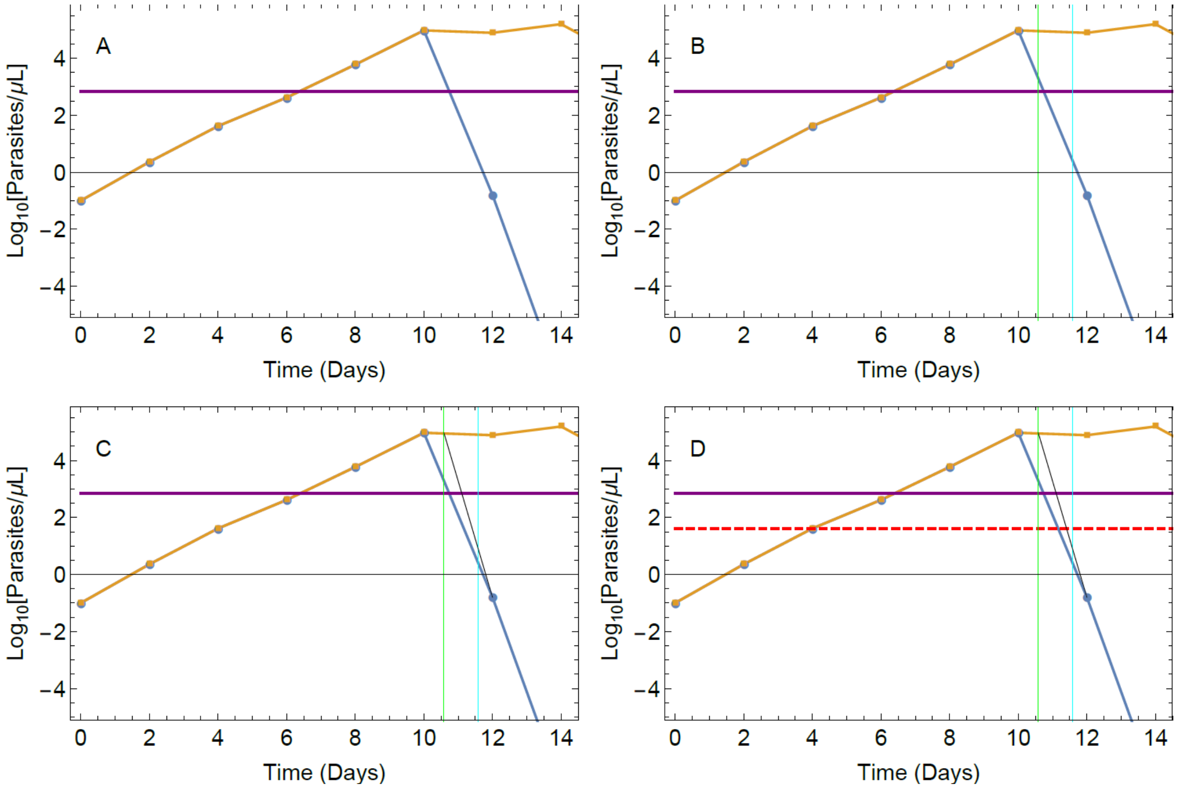

Supplementary Figure 4: The time series data recorded by the smart blister packs in Ref. (1) and model-estimated probability of parasitological treatment failure. Each row represents a patient, with each dose taken marked by a rectangle. Shorter rectangles indicate that the patient did not take the full dose. The rectangles are coloured to indicate the percentage of prescribed pills taken by the patient: all pills (black) between 80-99% (cyan), 60-79% (purple), 40-59% (orange), 20-39% (magenta), and <20% (brown). The vertical, red lines indicate the recommended timings of the six doses. For patients who took multiple pills per dose, we have grouped pills into doses if pills were taken within half an hour of each other. In this figure, patients are ordered by the probability that their adherence profile results in treatment failure, according to our within-host model. The probability of failing treatment was estimated from 10^4 simulations of the within-host model for each patient adherence profile. Doses taken after 75 hours are not shown here, but their effects are included in the model. Body weight data, needed to inform the PK model, were calculated as per Fig. 4 in the main text.

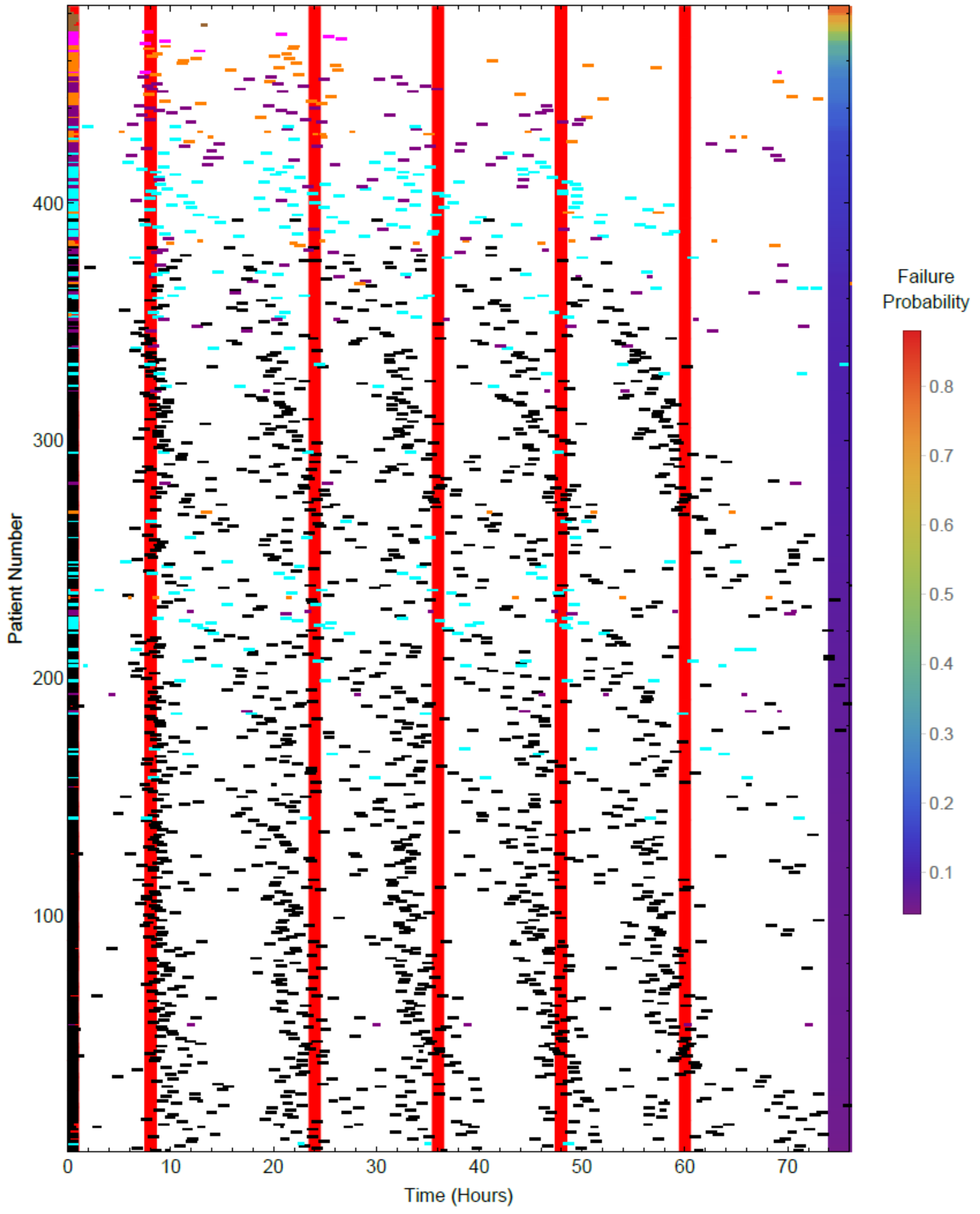

Supplementary Methods

Capturing the effective *var*-specific response (EVSR)

The efficacy of this immune response depends on which variants are present at a given time and on the previous exposure of the immune system to these variants. This averaged immunity can grow in strength, as the response to those particular variants improves, or it can wane, as known variants are removed and replaced with new, previously-unseen variants. In addition, this function should also reflect the fact that the efficacy of a *var*-specific immunity will wane over time if parasites stop expressing that *var* variant. However, the immune response to the return of a variant that has been present previously will be strengthened by immunological memory. This effect is explicitly included in the within-host model of Eckhoff (2). Furthermore, a model by Childs and Buckee introduces another immune function, describing a cross-reactive response(3). This means that antibodies produced in response to a particular variant have some efficacy against other, antigenically similar variants. We do not wish to explicitly include all of these factors, but our single function $S_v(t)$ must be able to represent their net effect.

Taking all these factors into account, we explored a variety of functions to describe an effective *var*-specific response, $S_v(t)$, which has some memory of past parasitaemia. The duration of the memory will increase slowly over the course of the infection. The intuitive motivation for doing this is as follows. Early on in the infection, the immune system of a naïve host will only have been exposed to a small number of *var* variants. In this instance, it should have a short memory: the introduction of a new variant will have the effect of weakening the immune response. Later on in the infection, however, when more of the variants have been present in the infection, we expect that antibodies to a wider repertoire of targets should be present. Therefore, a longer memory to previous parasitaemia gives a better guide to the efficacy of the EVSR.

We represent this memory as a function of the cumulative history of parasite density. At time t, we fix the upper limit of the sum to be equal to $t-4$ generations: this assumes that the antibody response to a particular target will take 8 days to appear. However, we allow the lower limit, denoted by the function $f(t)$, to vary over the course of the infection. Thus the EVSR can be represented by:

$$S_v(t) = \frac{1}{1 + \left(\frac{\sum_{\tau=f(t)}^{t-4} P(\tau)}{P_v^*} \right)^{\kappa_v}}. \qquad \text{(S1)}$$

Here the value of function $f(t)$ must be rounded to the nearest integer. When fitting the model, we examined three different choices for this function, and compared the best fit for each. The three functions were:

$$f(t) = \lambda^{(t-4)}(t-4), \qquad \text{(S2a)}$$

$$f(t) = (t-4)\exp[-(t-4)/c_0],\qquad \text{(S2b)}$$

$$f(t) = \frac{t-4}{1+\left(\frac{t-4}{c_1}\right)^{c_2}},\qquad \text{(S2c)}$$

where λ, c_0, c_1, and c_2 are constants. All functions have the same qualitative behaviour: the value of $f(t)$ remains close to the upper limit of the sum for small values of t, and the difference between the two limits grows as t increases. The two limits of the sum, which define the length of the memory in the EVSR, are illustrated in Supplementary Fig. 1 for one choice of $f(t)$.

Model fitting to the malaria therapy data

The malaria therapy dataset includes 334 adult male syphilis patients from the United States, with the majority reporting no previous exposure to malaria. Readings of asexual and sexual parasite densities were recorded daily, along with observations of fever. Parasitaemia was detected via microscopy with a detection threshold of approximately 10 PRBCs per microliter. For our analysis we used data from the subset of 35 patients who were classed as spontaneous cures and for whom sufficiently detailed follow up was done for a long duration. These patients were not discharged until they displayed negative results for a month of daily blood examination, followed by five months of blood examinations twice every week(4). For safety reasons, some patients received subcurative doses of antimalarial drugs during the infection. As these effects were seen to have limited and short lived effects(4), they will not be considered here. We note here that each patient was infected by a single strain of *falciparum* malaria. The strains used were: El Limon (17 cases), Santee Cooper (17 cases), and McLendon (1 case)(4).

Following the approach of Johnston et al, we compared the minimum, median and maximum values for key summary statistics for an ensemble of stochastic runs of the model to those calculated directly from the data. We fit our model to nine summary statistics: (i) the rate of growth of parasite density up until the first peak (a straight line on a log scale), (ii) the number of local peaks of parasite density, (iii) the slope of the best fit line joining the local maxima (iv) the log of the parasite density at the first peak, (v) the geometric mean of the time intervals between the local maxima, (vi) the last day a patient was parasite positive, (vii) the standard deviation of the logarithms of the time interval between consecutive local maxima, (viii) the proportion of parasite-positive observations in the first half of the interval between the first and last positive day, and (ix) the proportion of parasite-positive observations in the second half of the interval between the first and last positive day.

Here a local maximum was defined as a parasite density greater than the 3 previous observations and not less than the 3 following observations. We used base 10 for the logarithms of the parasite densities and took the threshold of detection via microscopy to be 10 PRBCs per μL. Therefore, when calculating statistics from the model simulations, we only used data points above this threshold.

To aid comparison with the results obtained by Johnston et al. we followed their sampling procedure(5). For each set of parameter values, an ensemble of 1000 trajectories were generated, and the nine summary statistics calculated. A bootstrapping procedure was then used, producing 50

samples of 50 stochastic realisations of the model. The minimum, median and maximum of each statistic was calculated for each sample. These values were then averaged over all 50 samples.

For each parameter set the model fit was evaluated by comparing the percentage differences between the model and the data i.e. $|(D-M)/D|$ where D represents the summary statistic from the data and M the one obtained from the model. There were 27 targets that informed the model: the minimum, median, and maximum of each summary statistic. A model employing a particular parameter combination was assessed using the sum of the percentage differences of 26 of these. The maximum value of the slope of the local maxima was excluded because its very small magnitude (see Table 1) led to large relative differences between the data and the model. This percentage difference dominated the other contributions, making it hard to assess the overall model fit.

Using the approach outlined above, we sought to find a minimum value for our objective function (the sum of the 26 percentage differences), by varying the model parameters. For our model, we found that stochastic variation in the model output translated into variation in the value of the objective function. By this we mean that generating two sets of 1000 simulations and performing the sampling described above can result in different values being found for the objective function. Therefore, we generated 100 batches of 1000 model runs, and used the median results obtained for each summary statistic to calculate the value of the objective function. MCMC methods were employed to generate random walks in parameter space, using the Metropolis-Hastings algorithm.

The two adaptive immune responses have a delayed response to parasitaemia, reflecting the time required to produce antibodies. This means that the size of the first peak is tightly controlled by the innate immune response. We retained the parameters used by Johnston et al. for the innate response, and varied the other parameters to obtain the best model fit. The parameters varied in the MCMC analysis were σ, g, k_m, P_v^*, as well as the parameter(s) defined in the function $f(t)$ (see Eq. 4). The remaining parameters were tuned by hand. The best-fit parameters are shown in Supplementary Table 1.

<u>The population pharmacokinetic model</u>

For a pharmacokinetic characterisation of artemether (AM) and lumefantrine (LMF) we employed a model due to Staehli-Hodel et al. (6). Their model, which importantly captures the observed population-level variation, was fitted to patients in Tanzania, who were positive by microscopy for *P. falciparum* malaria. For both drugs, a one-compartment PK model was fitted, with first-order absorption from the gastrointestinal tract (6). For both artemether and lumefantrine, concentrations of their active metabolite, dihydroartemisinin (DHA) and desbutyl-lumefantrine (DLF) respectively, was described via an additional compartment. Supplementary Fig. 2 shows the time-concentration profiles for LMF, DLF, AM and DHA. The three black lines indicate the 5th percentile, median, and 95th percentile of the drug concentration at each time point. Here we simulated a cohort with normally distributed body weights, with $\mu = 11.1$ and $\sigma = 2.8$ (in kilograms), truncated so that the minimum possible weight was 5kg. This matches the body weight distribution used to fit the PD model.

Describing the waiting time for obtaining antimalarials

Once the pharmacokinetic model is characterised, one must decide at what point during the infection the treatment should begin. We break this down into two questions: (i) at what parasite density does fever break, and (ii) how do we characterise the waiting time between the onset of symptoms and treatment commencing? To answer the first question, we turn again to the analysis of the malaria therapy dataset. In a previous modelling study by Dietz et al.(7), the pyrogenic threshold (parasite density at which fever is triggered) was compared with the peak parasitaemia observed ($P_{\max}$). A wide variation in behaviour was observed. The authors described this variation by a random variable d, drawn from a uniform distribution U(-3.699,0). The pyrogenic threshold was related to the peak parasitaemia by the relation $10^{d} \times P_{\max}$. Since the parasite peak here relates to the untreated infection we also run the model without treatment to calculate $P_{\max}$ (see Supplementary Fig. 3).

To assess typical waiting times for treatment we used data collected by the DHS (Demographic and Health Surveys) Program (8). We fitted a continuous distribution to data using a maximum likelihood approach. This data is presented as the number of days each patient surveyed waiting for treatment and is integer valued. We fitted a continuous-time distribution to this data by calculating the maximum likelihood of binned data for a number of different distributions. If we write n_c as the number of data points present in bin $[m_c, m_c + 1)$ then the likelihood of a distribution characterised by parameters θ is

$$L(\theta \mid D) = \prod_{c} (P(m_c \le X \le m_c + 1 \mid \theta))^{n_c} . \qquad \text{(S3)}$$

This is equivalent to

$$L(\theta \mid D) = \prod_{c} (F(m_c + 1 \mid \theta) - F(m_c \mid \theta))^{n_c} . \qquad \text{(S4)}$$

We calculated maximum-likelihood estimates for several two-parameter distributions: log normal, Weibull and gamma. According to the maximum likelihood obtained for each distribution, the best fit was the log normal distribution, with parameters $\mu = 0.70$ and $\sigma = 0.6575$. Since, in each case, only two parameters were varied, the parameter space was simply explored by moving over points on a regular lattice.

So, to summarise, two random numbers must be drawn to determine the timing of the first dose of AL: one to determine a patient's pyrogenic threshold, and another to return the subsequent waiting time for obtaining treatment. The dose of AL each patient received depends on their body weight, as described in the Results section.

Combining the PKPD & parasitaemia models

In our model, we assume that the antimalarial drugs act independently of each other e.g. there are no synergistic or antagonistic effects. Our PD model can be written in the following form:

$$\frac{dP}{dt} = -\left[k_{\max}^{AM} \frac{C_{AM}(t)}{C_{AM}(t) + C_{50}^{AM}} + k_{\max}^{DHA} \frac{C_{DHA}(t)}{C_{DHA}(t) + C_{50}^{DHA}} + k_{\max}^{LMF} \frac{C_{LMF}(t)}{C_{LMF}(t) + C_{50}^{LMF}} \right] P. \tag{S5}$$

Where the concentration of each drug or metabolite as a function of time is defined by the PK model. To simplify the notation, we rewrite this as

$$\frac{dP}{dt} = -\left[h_{AM}(t) + h_{DHA}(t) + h_{LMF}(t) \right] P. \tag{S6}$$

As stated in the text, we have here neglected the antimalarial action of the metabolite DLF. We now must combine this PD model with the discrete-time model for asexual parasitaemia. We write the effects of the drugs over a 48 hour time period as:

$$P(t = 48) = \left(\exp\left[-\int (h_{AM}(t) + h_{DHA}(t) + h_{LMF}(t))dt \right] \right) P(t = 0) \equiv f \times P(t = 0). \tag{S7}$$

Therefore, we build up the effects of the antimalarial drugs over a 48 hour period, and then combine with the parasitaemia model. Now expressing time in units of 48 hours we write

$$P(t+1) = f \times m(t)[S_c(t) S_v(t) S_g(t)] P(t) . \tag{S8}$$

Here f represents the parasite killing carried out over the 48 hour interval. Clearly, until the first dose is administered $f = 1$ as expected. This procedure is then repeated, until either the effects of the drugs or the immune system (or a combination of both) drives the parasite density below the cut-off density, and the infection is deemed to have ended.

Fitting the PD model

We fitted the parameters in the PD model (see Eq. S5) by observing the effects of the antimalarial drugs on the parasitaemia or, more specifically, the effects on patient outcomes. We examine the number of treatment failures, which we measure as the proportion of patients who were parasite positive by microscopy 28 days after treatment commenced. We also examined how quickly parasites were cleared after the first dose was administered, by looking at the proportional of patients who were still parasite positive one and two days after the start of treatment. Data from these come from a large trial of ACTs administered to African children following episodes of uncomplicated episodes of P. falciparum malaria. The study included several sites, with a range of transmission intensity. We focused on low transmission settings, to minimise the effects that previous malaria episodes would have on the immune response.

Repeated stochastic simulation of our model produces variation in the results obtained. To match the Ndola cohort who were treated with AL, we repeatedly simulated cohorts of 75 patients and for each cohort recorded the proportion of patients who were parasite positive on day 1 and day 28. To calculate the former, we use log-linear interpolation, as illustrated in Supplementary Fig, 3. So, for each parameter combination, λ , we obtain a histogram of outcomes for each statistic. We use these to approximate the probability distribution for observing, the outcomes for the two statistics, say S , and therefore the probability of obtaining the observed results, S^{obs} , which we write as $P(S^{obs} \mid \lambda)$. This approach is illustrated in Fig. 5b in Ref. (9), and is sometimes referred to as the pseudo (or synthetic) likelihood approach.

By neglecting the effects of metabolite DLF and stipulating that AM and DHA have the same PD parameters, we are left with four parameters to fit (see Eq. S5 and Table 2). We used information on the in vitro properties of the drugs and estimates of the parasite reduction ratio(10) to constrain the parameter ranges explored.

Supplementary References

1. Bruxvoort K, Festo C, Cairns M, Kalolella A, Mayaya F, Kachur SP, et al. Measuring Patient Adherence to Malaria Treatment: A Comparison of Results from Self-Report and a Customised Electronic Monitoring Device. PLoS One. Public Library of Science; 2015 Jan 1;10(7).
2. Eckhoff P. P. falciparum Infection Durations and Infectiousness Are Shaped by Antigenic Variation and Innate and Adaptive Host Immunity in a Mathematical Model. PLoS One. Public Library of Science; 2012 Jan 1;7(9).
3. Childs LM, Buckee CO. Dissecting the determinants of malaria chronicity: why within-host models struggle to reproduce infection dynamics. J R Soc Interface. The Royal Society; 2015 Mar 6;12(104):20141379.
4. Molineaux L, Diebner HH, Eichner M, Collins WE, Jeffery GM, Dietz K. Plasmodium falciparum parasitaemia described by a new mathematical model. Parasitology. Cambridge University Press; 2001 Apr 1;122(4):379–91.
5. Johnston GL, Smith DL, Fidock DA. Malaria's Missing Number: Calculating the Human Component of R-0 by a Within-Host Mechanistic Model of Plasmodium falciparum Infection and Transmission. PLOS Comput Biol. Public Library of Science; 2013 Apr 1;9(4).
6. Hodel EMS, Guidi M, Zanolari B, Mercier T, Duong S. Population pharmacokinetics of mefloquine, piperaquine and artemether-lumefantrine in Cambodian and Tanzanian malaria patients. Malar J. 2013 Jan 1;
7. Dietz K, Raddatz G, Molineaux L. Mathematical model of the first wave of Plasmodium falciparum asexual parasitemia in non-immune and vaccinated individuals. Am J Trop Med Hyg. American Society of Tropical Medicine and Hygiene; 2006 Aug 1;75(2):46–55.
8. Measure-DHS [Internet]. Demographic Health Surveys. Available from: http://measuredhs.com/
9. Hartig F, Calabrese JM, Reineking B, Wiegand T, Huth A. Statistical inference for stochastic simulation models – theory and application. Ecol Lett. Blackwell Science Ltd; 2011 Aug 1;14(8):816–27.
10. White NJ. Assessment of the pharmacodynamic properties of antimalarial drugs in vivo. Antimicrob Agents Chemother. American Society for Microbiology; 1997 Jul 1;41(7):1413–22.
